# Advancements in the development of antivirals against SARS-Coronavirus

**DOI:** 10.3389/fcimb.2025.1520811

**Published:** 2025-01-23

**Authors:** Mrityunjay Kumar, Mirza Sarwar Baig, Kanchan Bhardwaj

**Affiliations:** ^1^ Department of Biotechnology, School of Engineering and Technology, Manav Rachna International Institute of Research and Studies, Faridabad, India; ^2^ Centre for Virology, School of Interdisciplinary Science and Technology, Jamia Hamdard, New Delhi, India; ^3^ Regional Centre for Biotechnology, NCR Biotech Science Cluster, Faridabad, India

**Keywords:** COVID-19, long COVID, SARS coronavirus, betacoronavirus, antivirals

## Abstract

Severe Acute Respiratory Syndrome Coronavirus (SARS-CoV) caused an outbreak in 2002-2003, spreading to 29 countries with a mortality rate of about 10%. Strict quarantine and infection control methods quickly stopped the spread of the disease. Later research showed that SARS-CoV came from animals (zoonosis) and stressed the possibility of a similar spread from host to human, which was clearly shown by the COVID-19 outbreak. The COVID-19 pandemic, instigated by SARS-CoV-2, has affected 776 million confirmed cases and more than seven million deaths globally as of Sept 15, 2024. The existence of animal reservoirs of coronaviruses continues to pose a risk of re-emergence with improved fitness and virulence. Given the high death rate (up to 70 percent) and the high rate of severe sickness (up to 68.7 percent in long-COVID patients), it is even more critical to identify new therapies as soon as possible. This study combines research on antivirals that target SARS coronaviruses that have been conducted over the course of more than twenty years. It is a beneficial resource that might be useful in directing future studies.

## Introduction

**Figure 1:**
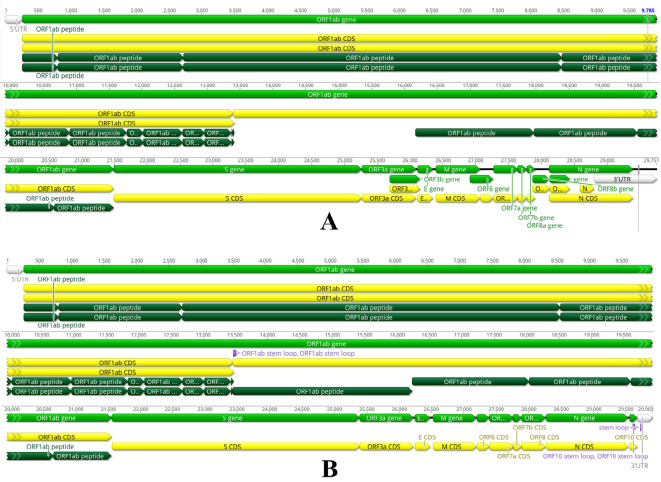
SARS-CoV-1 and SARS-CoV-2 belong to the genus betacoronavirus, showing notable differences in genomic sizes and features. **(A)** SARS-CoV-1 genome size is 29751 bases, **(B)** SARS-CoV-2 genome size is 29903 bases, slightly bigger than CoV-1. Both are positive-sense, single-stranded RNA (+ssRNA) and share a similar genomic architecture, including a 5’ untranslated region (UTR), coding regions (CDS) for nonstructural and structural proteins, and a 3’ untranslated region (UTR). Both genomes have a similar organisation, comprising around fourteen open reading frames (ORFs) encoding twenty-seven proteins. The genes are organised in the following order: 5’ UTR – ORF1ab (nonstructural proteins) – structural proteins (S, E, M, N) – 3’ UTR. The largest ORF, ORF1ab (light green), occupies two-thirds of the genome and encodes a polyprotein processed into sixteen nonstructural proteins (NSPs). ORF1ab CDS (yellow) indicates the coding sequence for the ORF1ab polyprotein, with regions labelled as ORF1ab peptide (dark green), denoting cleavage sites or functional peptides derived from the polyprotein. Both viruses have conserved structural protein genes encoding the envelope (E), spike (S), membrane (M), and nucleocapsid (N) proteins. Both viruses utilise a discontinuous transcription mechanism, which generates a set of subgenomic mRNAs. These subgenomic mRNAs are used for the translation of structural and accessory proteins. The transcriptional regulatory sequences (TRSs) upstream of each gene share similar structures in both viruses, allowing efficient transcription. SARS-CoV-1 and SARS-CoV-2 encode small accessory ORFs, such as ORF3a, ORF6, ORF7a, ORF7b, and ORF8. These smaller ORFs, located between structural protein genes, encode accessory proteins that modulate host immune responses and may contribute to immune evasion, host interactions, and virus replication. The ORF8 gene has undergone major changes between the two viruses. It is established that ORF8 in SARS-CoV-1 encodes two distinct accessory proteins, 8a and 8b, while a single accessory protein is produced by SARS-CoV-2 ORF8. Interestingly, SARS-CoV-2 has an additional ORF10 adjacent to the 3’ UTR encoding a single accessory protein. Meanwhile, the SARS-CoV-2 genome encodes characteristic “stem-loop” structures marked within ORF1ab, ORF10 and 3’UTR, which could represent a regulatory RNA secondary structure essential for viral replication and transcription.

SARS-CoV-2, a betacoronavirus, emerged in Wuhan, China, in December 2019, causing a respiratory syndrome, COVID-19, which became a pandemic by March 2020 (Chan et al., 2020; Huang et al., 2020). Since then, it has spread to 206 countries and territories, causing 776 million confirmed cases and more than seven million deaths globally as of Sept 15 2024 (World Health Organization (WHO), 2024). COVID-19, caused by SARS-CoV-2, follows past outbreaks of related coronaviruses, including MERS-CoV in 2012 and SARS-CoV in 2003. SARS-CoV-2 and SARS-CoV share significantly high genomic similarity. Although, notable differences at specific amino acid residues in key proteins also exist (**Figure 1**). Despite high similarity, SARS-CoV-2’s global impact has been far more widespread and devastating (Baig et al., 2021; Gorkhali et al., 2021; Baig et al., 2023). However, due to the similarities among the two coronaviruses, antiviral research involving SARS-CoV has become relevant for SARS-CoV-2, as described in the following sections.

## Timeline of antiviral therapies for SARS coronaviruses

Over the past two decades, antiviral therapy research for SARS-CoV has progressed dramatically. The COVID-19 pandemic began in 2020, rapid advancements were made with drugs like Remdesivir and Favipiravir, alongside continued investigation into other repurposed and novel therapies. Various synthetic and natural compounds have been used to treat coronavirus infections throughout previous pandemics and early epidemics from 2003 to 2024. This paper provides a thorough evaluation of these compounds.

### 2003-2006

The 2003 SARS outbreak prompted quick research and evaluation of various existing pharmaceuticals. Because of their antiviral properties, chloroquine and nicosamide, initially prescribed as antimalarial and antihelminthic medications, respectively, were repurposed (Cinatl et al., 2003). The potential of luteolin, a naturally occurring flavonoid, glycyrrhizin, hexachlorophene, vanillinbananin, and alpha-Hederin to prevent SARS-CoV replication was investigated. Early attempts to diversify antiviral options were also made with the testing of saponins, valinomycin, and promazine (Cinatl et al., 2003). Another significant discovery from 2003 was the repurposing of penicillium-derived antibiotic mycophenolic acid (MPA), which has shown promise in preventing or minimising coronavirus infection. In 2004, further research led to the discovery of Beta-D-N4-Hydroxycytidine (NHC), which inhibited HCoV-NL63 (EC50 of 400 nM and CC50 of >100 μM) and SARS-CoV (EC50 of 10 μM and CC50 of >100 μM) (Barnard et al., 2004b). Also, an antihelminthic drug, Niclosamide, showed SARS-CoV inhibition at post-entry steps in Vero cells (EC50 of 1–3 μM and CC50 of 250 μM) (Wu et al., 2004). N-(2-aminoethyl)-1 aziridineethanamine (NAAE), a new small-molecule inhibitor (EC50 of 0.5 μM), represented a big step in targeting the SARS-CoV spike protein-mediated cell fusion capabilities (Huentelman et al., 2004). Researchers began to focus on structurally customised inhibitors, such as 2-acetamido-alpha-D- derivatives (Hoever et al., 2005) and Phe-phe-dipeptide inhibitor c(JMF 1521) (Shie et al., 2005), which emerged as potent inhibitors. 2-acetamido-α-D-glucopyranosylamine, benzylcysteine, and Gly-Leu peptide cause an increase of 10-fold to 70-fold in anti-SARS-CoV activity by inhibiting viral entry. Ribavirin (nucleoside analogue of guanosine), which has historically been used to treat Hepatitis C, was tested for its antiviral activity against SARS-CoV *in vitro* (EC50 of 20 μg/mL and CC50 of >200 μg/mL) (Saijo et al., 2005). Adamantane-based compounds such as Bananins, specifically engineered to inhibit helicase activities and replication of SARS Coronavirus (EC50 of <10 μM and CC50 of 390 μM), were also introduced as promising antiviral agents (Tanner et al., 2005). The emphasis on derivatives remained in 2006; for example, the Eremomycin derivative molecules 27 and 39 (Gan et al., 2006) showed efficacy in blocking viral proteases SARS-CoV (EC50 of 14 μM and CC50 of 22 μM) (Balzarini et al., 2006). In contrast, pyrimidine analogue 6-azauridine emerged as a nucleoside analogue for suppressing HCoV-NL63 replication (EC50 of 32 nM and CC50 of 80 μM) (Pyrc et al., 2006). The antiviral effects of mycophenolic acid (MPA) and ribavirin were confirmed by further characterisation (Barnard et al., 2006). One of the most important factors in preventing viral infections is the suppression of immunological responses, which MPA blocks *in vivo* by suppressing the apoptosis-inducing enzyme IMP dehydrogenase in the host (BALB/c mice) (Barnard et al., 2006) (**Table 1**).

**Table 1:** Year-wise compilation of coronavirus inhibitors.

Year	Inhibitors and drugs	References
2003	Chloroquine, Luteolin, Hexachlorophene, Vanillinbananin, Glycyrrhizin, alpha-Hederin, Saponins, Valinomycin, Niclosamide, Promazine, Mycophenolic acid	(Cinatl et al., 2003)
2004	Beta-D-N4-Hydroxycytidine, Niclosamide, N-(2-aminoethyl)-1 aziridineethanamine (NAAE)	(Barnard et al., 2004a; Huentelman et al., 2004; Wu et al., 2004)
2005	2-Acetamido-alpha-D-derivative, Phe-phe-dipeptide inhibitor c (JMF 1521), Ribavirin, Bananins	(Hoever et al., 2005; Saijo et al., 2005; Shie et al., 2005; Tanner et al., 2005)
2006	Eremomycin derivative, 6-azauridine, Ribavirin, Mycophenolic acid	(Balzarini et al., 2006; Barnard et al., 2006; Gan et al., 2006; Pyrc et al., 2006)
2007	Ribavirin, Emodin,	(Åkerström et al., 2007)
2008	Lopinavir, ritonavir, Curcumin	(Kutluay et al., 2008)
2010	Benzopurpurin B, C-473872, Tetrahydroquinoline Oxocarbazate (CID-23631927), TAPI2, 2-(benzylthio)6-oxo-4-phenyl 1-6 dihydropyrimidine, Griffithsin	(Haga et al., 2010; Ortiz-Alcantara et al., 2010; Ramajayam et al., 2010; Shah et al., 2010)
2011	Mucroporin	(Li et al., 2011)
2012	Scutellarin, dipeptidyl aldehyde (GC373), dipeptidyl bisulphite salt (GC376)	(Kim et al., 2012; Struck et al., 2012; Yu et al., 2012)
2013	Cyclosporin, N-(9,10-dioxo-9,10-dihydroanthracen-2-yl)benzamide (SSAA09E1), N-[[4-(4-methylpiperazin-1-yl)phenyl]methyl]-1,2-oxazole-5-carboxamide (SSAA09E2), [(Z)-1-thiophen-2-ylethylideneamino]thiourea (SSAA09E3)	(Adedeji et al., 2013; De Wilde et al., 2013)
2014	Mycophenolic acid, Arbidol	(Blaising et al., 2014; Hart et al., 2014)
2015	Acyclovir, Tat-P29 (TP29)	(Peters et al., 2015; Wang et al., 2015)
2016	Nitazoxanide, Tizoxanide, Favipariavir, Ribavirin, Remdesivir, Lopinavir-Ritonavir, Nafamostat, Myricetin,	(Dorman and Dorman, 2016; Rossignol, 2016; Sissoko et al., 2016; Warren et al., 2016; Yamamoto et al., 2016; Zumla et al., 2016)
2017	Alisparivir, Favipariavir, Remdesivir, Saracatinib	(De Wilde et al., 2013; Furuta et al., 2017; Sheahan et al., 2017)
2018	Saracatinib, Disulfiram, Deguelin	(Lin et al., 2018; Nukui et al., 2018; Shin et al., 2018)
2019	Interferon therapy, Favipariavir, Remdesivir	(De Clercq, 2019; Li et al., 2019; Mulangu et al., 2019; Wang et al., 2020)
2020	Glycyrrhizic acid, Garcinolic acid, Lobaric acid, Tirilazad, Montelukast, Beta-D-N4-Hydroxycytidine, Remdesivir, Chloroquine (CQ), Hydroxychloroquine, Oseltamivir, Ribavirin, Arbidol Hydrochloride (Umifenovir), Favipiravir, Betulinic Acid, Peptidomimetc, Ebselen, Rivavirin, Octasaccharide, Hexamethylene, Camostat, Imanitib, Daenorubicin, Valproic acid, Silmitasertib, Ledipsavir, Darunavir, Saquinavir, Velpatasvir, Macaflavanone, Vibsanal, Belachinal, Ponatinib, Migalastat, Azithromycin, Ivermectin, Fluvoxamine, Lopinavir/Ritonavir, Umifenovir, Corticosteroid (Dexamethasone), Tocilizumab, Convalescent plasma, HCoV-OC43-HR2-derived peptide EK1, and its lipopeptide EK1C4, Arbidol, Galidesivir, EIDD-2801 (Molnupiravir or MK-4482), Disulfiram, Carmofur, Glycyrrhizin, Nobiletin, Neohesperidin, SSAA09E2, Chlorpromazine, Camostat Mesylate, Nafamosta, nitazoxanide, Interferon alfa-2a and -2b, Deguelin, Apilimod	(Caly et al., 2020; Cao et al., 2020; Chen et al., 2020; Dai et al., 2020; Elfiky, 2020; Ferner and Aronson, 2020; Glebov, 2020; Hoffmann et al., 2020; Huang et al., 2020; Jin et al., 2020; Liao et al., 2020; Ma et al., 2020; Oany et al., 2020; Oroojalian et al., 2020; Riva et al., 2020; Sanghai and Tranmer, 2020; Santos et al., 2020; Shalayel et al., 2020; Sheahan et al., 2020; Sinha et al., 2020; Stone et al., 2020; Tiwari et al., 2020; Touret and De Lamballerie, 2020; Wang et al., 2020; Zeng et al., 2020; Zhou et al., 2020; Afsar et al., 2022)
2021	Molnupiravir (MK-4482 or EIDD-2801), Barrigenol, Kaempferol, Myricetin, Inarigivir soproxil, Foretinib, Eprosartan, Darunavir, Camostat Mesylate, Ivermectin, nitazoxanide, Interferon alfa -2a and -2b, Deguelin, Nilotinib, Sorafenib, Saquinavir, Aprepitant, Valrubicin, imatinib, Mycophenolic acid, Quinacrine Dihydrochloride, Monoclonal antibodies (P2C-1F11, P5A-1D2, P5A-3C8, P22A-1D2, P5A-1B9 and P2B-1G5), Antibodies (REGN10933, REGN10987, CB6, S309, 4A8 and CR3022), A human monoclonal antibody (JMB2002), Dexamethasone, Sarilumab, Natural compounds PubChem IDs (1777791, 95372568, 25575299, 1776037, 1751157), sarsasapogenin, ursonic acid, Silymarin, Novobiocin, Aranotin, Ajmalicine, ritonavir, remedesivir, oseltamivir, lopinavir, favipiravir, Acarbose, Cefotiam, Ceforanide, Thymopentin, Oleuropenin, Ginsenoside, Kaempferitrin, Rhoifolin, Lopinavir and Rivabirin, Isoliquiritinapioside, Liquiritin, Dehydroglyasperin C, Casirivimab + Imdevimab, Bamlanivimab + Etesevimab, Cilgavimab + Tixagevimab, Sotrovimab, Regdanvimab, Bamlanivimab Monotherapy, Bamlanivimab + Etesevimab, Casirimab + Imdevimab, Sotrovirmab, Nafamostat, Camostat, Apilimod, Chloroquine, Hydroxychloroquine, Niclosamide, Clofazimine, Nitazoxanide, Emetine, Ivermectin, Ciclesonide, Fluvoxamine, digoxin, remdesivir, dronedarone, atovaquone, mebendazole, ouabain, entacapone, Phenformin, Nilotinib, Lumacaftor, Rimegepant, Dihydroergotamine, Conivaptan, Roferon, Wellferon, Alferon, Rebif, Betaferon, Acyclovir, Ganciclovir, Ribavirin, Indinavir (Crixivan), Nelfinavir (Viracept), Saquinavir (Fortovase), Lamivudine (Epivir), Zidovudine (Retrovir), Neuroaminidase inhibitors, Oseltamivir (Tamiflu), Zanmivir (Relenza), Amantadine (Symmetrel), Foscarnet (Foscavir)	(Atyeo et al., 2021; Batool et al., 2021; Breining et al., 2021; Castelnovo et al., 2021; De Lima Menezes and Da Silva, 2021; Gu et al., 2021; Han et al., 2021; Joshi and Poduri, 2021; Kumar et al., 2021; Mahmud et al., 2021; Rajter et al., 2021; Sharma et al., 2021; Sinha et al., 2021; Solidarity Trial Consortium, 2021; Sun et al., 2021; Tang et al., 2021; The RECOVERY Collaborative Group, 2021; Vijayan and Gourinath, 2021; Weinreich et al., 2021; Khan et al., 2022; Scholl et al., 2022; Sharma et al., 2022)
2022	Nirmatrelvir (CID-155903259), Remdesivir (CID-121304016), Ritonavir, Inotodiol (CID-182264), Neosarcodonin A, Cyathatriol, Cyathin-B3, Erinacine A, Lucidadiol, Enolipodin D, Ganodermadiol, Sarcodonin, Coprinol, Sotrovimab, Bebtelovimab, Tixagevimab + Cilgavimab, Bamlanivimab + Etesevimab, Casirivimab + Imdevimab, Molnupiravir, Paxlovid (Nirmatrelvir/Ritonavir), Dutasteride, Etoposide, Golvatinib, Irinotecan, Meprednisone, Tasosartan, Simeprevir (DB06290), Paritaprevir (DB09297), Artesunate (DB09274), Naldemedine, Ergotamine, Simvastatin), Telmisartan (ZINC000001530886), Cephalexin, Olmesartan, Dihydrocodeine, Klonopin, ZINC000255977092, ZINC000257362202, Diosgenin glucoside (Diaglucide), Alpha-Ergocryptine, Capmatinib, Lixivaptan	(Al-Rashedi et al., 2022; Barage et al., 2022; Gottlieb et al., 2022; Khan et al., 2022; Ng et al., 2022; Saramago et al., 2022)
2023	Cafestol, kahweol, theaflavin 3,3′-digallate, Dihydroergocristine, Ergometrine, Ubidecarenone, Tacrine, Perflubron, Rasagiline, Talastine, Barnidipine, Glasdegib, Lercanidipine, Tipranavir, Temoporfin, Irinotecan, Zorubicin, Ciclesonide, Risdiplam, Ledipasvir, Suramin, Venetoclax, Remdesivir, Paxlovid, Molnupiravir, Nitazoxanide, Favipiravir, Lopinavir/ritonavir, Chloroquine and hydroxychloroquine, Ivermectin, bamlanivimab/etesevimab, casirivimab/imdevimab, tixagevimab/cilgavimab, Monoclonal antibodies (mAbs), sotrovimab, bebtelovimab, Sotrovimab, cilgavimab, Interleukin-6 inhibitors with Tocilizumab and Sarilumab, Anakinra, Baricitinib, Tofacitinib, Vilobelimab, Colchicine, Conakinumab, Dexamethasone, Cortisone, Atovaquone, Pibrentasvir + Oritavancin, Ledipasvir, Micafungin, Linaclotide + Desmopressin, Vitamin B_12_ (methylcobalamin and cyanocobalamin)	(Aboul-Fotouh et al., 2023; Dhanabalan et al., 2023; Jamir et al., 2023; von Beck et al., 2023)
2024	Tocilizumab, anakinra, vilobelimab, baricitinib, Paxlovid (nirmatrelvir/ritonavir), remdesivir, molnupiravir, pemivibart, Anti-inflammatory corticosteroids-dexamethasone, bamlanivimab/etesevimab, Actemra, sotrovimab, Kineret, Ensitrelvir.	(Stone et al., 2020; Gottlieb et al., 2022; Yamato et al., 2024)

Co-packaged medicines (/) and combination therapy (+) are especially highlighted.

### 2007-2010

Repurposing has been a significant trend throughout the years. A reevaluation of the effectiveness of ribavirin and emodin was performed, with an exploration of the prospect of interferon therapy to augment immune responses against the virus (Åkerström et al., 2007). Ritonavir and Lopinavir were part of the combo therapies offered for SARS-CoV in 2008. Curcumin also became a contender because of its antiviral and anti-inflammatory qualities (Kutluay et al., 2008). By 2010, several novel compounds were introduced, such as Benzopurpurin B, C-473872 and Tetrahydroquinoline Oxocarbazate (Ortiz-Alcantara et al., 2010). A plant-derived protein, Griffithsin, has shown strong efficacy in blocking viral entry (O’Keefe et al., 2010). It became clear that there was a paradigm shift toward exploring remedies that were more natural or drawn from biological sources. SARS-CoV and SARS-CoV-2 are constantly developing, and researchers are continuing their search for effective antiviral medicines. A significant focus exists on both synthetic and natural compounds that may enhance efficacy against the SARS-CoV and hCoV-2 strains (**Table 1**).

### 2011 to 2019

Between 2011 and 2019, the primary emphasis was on the development of new chemicals. This time signified substantial advancements in the comprehension of coronavirus pathophysiology and viral replication processes. This comprehension establishes a vital basis for formulating therapies for the ensuing SARS-CoV-2 epidemic. In 2011, researchers discovered mucroporin-M1, an antimicrobial peptide (AMP) exhibiting antiviral efficacy against the influenza virus (H5N1), measles virus (MeV), and SARS-CoV, signifying a potential foundation for further development (Li et al., 2011). They demonstrated that mucroporin-M1 could inhibit SARS coronavirus entry in HeLa-ACE2 cells with an EC50 of 14.46 μg/mL and a CC50 of 61.58 μg/mL with intense virucidal activity (Li et al., 2011).

By 2012, the antiviral focus had expanded to include molecules such as Scutellarin and Tyr-lys-Tyr-Arg-Tyr-Leu, hexapeptides that showed inhibitory activity against SARS-CoV, HCoV-NL63, and SARS-CoV-2 infection in Vero cells without any cytotoxic effects (Struck et al., 2012). The discovery of protease inhibitors, such as dipeptides dipeptidyl aldehyde (GC373) and dipeptidyl bisulphite adduct salt (GC376), was particularly significant as it targeted the SARS-CoV 3CLpro, with IC50 of 3.48 and 4.35 μM, respectively (Kim et al., 2012). In 2013, three derivatives of N-(9,10-dioxo-9,10-dihydroanthracen-2-yl)benzamide (SSAA09E1), N-[[4-(4-methylpiperazin-1-yl)phenyl]methyl]-1,2-oxazole-5-carboxamide (SSAA09E2), and [(Z)-1-thiophen-2-ylethylideneamino]thiourea (SSAA09E3) were developed. These inhibitors showed activity against SARS-CoV by blocking virus-host cell early interactions at EC50 varying from 3.1-9.7 μM and CC50 varying from 20- 100 μM (Adedeji et al., 2013). Additionally, a peptide, Cyclosporin A, was recognised for its broad-spectrum ability to inhibit diverse SARS-CoV, HCoV-229E and MHV replication or transcription, furthering the exploration of immunosuppressive drugs in antiviral therapy (De Wilde et al., 2011; Pfefferle et al., 2011; De Wilde et al., 2013). 2014 saw the re-emergence of Mycophenolic acid (MPA), a penicillium-derived antibiotic traditionally used as an immunosuppressant. MPA also inhibited MERS-CoV replication in Vero cells with an EC50 of 2.87 μM (Hart et al., 2014). Another compound, Arbidol and its derivatives, a broad-spectrum antiviral used in the treatment of influenza viruses, demonstrates the potential to reduce the SARS-CoV viral load *in vitro* at 50 μg/mL (Blaising et al., 2014; Hart et al., 2014).

This indicates a growing focus on utilising drugs that have already demonstrated safety for the treatment of SARS-CoV. In 2015, an innovative acyclic sugar scaffold of acyclovir was introduced, demonstrating structural modifications that improved its antiviral properties against coronaviruses, specifically MERS-CoV (EC50 and CC50 of 23 and 71 μM) and HCoV-NL63 (EC50 and CC50 of 8.8 and 120 μM) (Peters et al., 2015). A significant advancement was HIV Tat-protein-derived peptide (YGRKKRRQRRRGSG) known as Tat-P29 (TP29), which demonstrated strong broad-spectrum antiviral activity, effectively inhibiting *in vitro* replication of SARS-CoV (with an EC50 of 200 μM) and MHV (with an EC50 of 60 μM) (Wang et al., 2015). TP29 negatively affected the 2’-O-MTase activity of nsp10/nsp16, disrupting the replication process of the SARS-CoV genome.

Broad-spectrum coronavirus antiviral medication research advanced in 2016 with numerous promising candidates. Myricetin, a flavonoid, inhibited HCoV-229E and SARS-CoV-2 Mpro and 3CL-Pro enzymes, preventing *in vitro* replication (Dorman and Dorman, 2016). Nitazoxanide and its derivative, Tizoxanide, displayed broad-spectrum antiviral activities exploited for treating influenza viruses (A and B), as well as Ebola virus (EBOV) (Rossignol, 2016). *In vitro* studies on LLC-MK2 cells demonstrated that Nitazoxanide could inhibit MERS-CoV with an EC50 of 0.92 μg/mL. Favipiravir, Ribavirin, and Remdesivir have shown effectiveness in inhibiting viral RNA polymerase, indicating significant potential for treating coronavirus (Sissoko et al., 2016). The protease inhibitors Lopinavir-Ritonavir and Nafamostat demonstrated efficacy in inhibiting viral replication by acting on the viral protease (Zumla et al., 2016; Yamamoto et al., 2020).

In 2017, researchers continued to look for antiviral drugs that may stop coronaviruses from replicating. Remdesivir maintained its encouraging performance in preventing viral replication in both *in vitro* and animal models, whereas Alisparivir and Favipiravir were investigated further for their ability to target RNA polymerase (Furuta et al., 2017; Sheahan et al., 2017). In 2018, the identification of drugs like Saracatinib, a kinase inhibitor, and Disulfiram, typically used for alcohol dependence, broadened the range of repurposed drugs with possible antiviral activity (Shin et al., 2018). Deguelin (9,10-Dimethoxy-3,3-dimethyl-13,13a-dihydro-3H-chromeno[3,4-b]pyrano[2,3-h]chromen-7(7aH)-one) is a natural compound known for its ability to inhibit the HCV replication mechanism. Its potential application in combating SARS-CoV infection underscores the importance of plant-based antivirals (Nukui et al., 2018).

In 2019, these efforts reached a significant point with the additional use of Remdesivir (a nucleoside analogue- GS-5734) and Favipiravir (a purine nucleic acid analogue- T-705). Both compounds demonstrated notable potential in clinical trials and emerged as essential instruments in combating coronaviruses (Mulangu et al., 2019; Declercq et al., 2022). Exploring interference techniques such as interference RNAs (iRNAs) and short interference RNAs (siRNAs) for the treatment of HBV, HCV, HIV, and HTLV infections (Hernández and Sanan-Mishra, 2017) was also found to be a promising antiviral against structural proteins E, M, and N of SARS-CoV. This technique is also potent in antiviral therapies by modulating the host immune response (Li et al., 2011). Overall, the period from 2011 to 2019 laid a strong foundation for developing effective antiviral drugs against SARS-CoV. From 2003 to 2019, a heap of data of *in vitro* and *in vivo* antiviral activity assays for several molecules against CoVs was generated. It was high time to critically compare these molecules that could be further investigated for their clinical applicability. The research during these years expanded the antiviral arsenal and provided evidence of the mandatory need for further research and rapid development of broad pharmaceutical agents against emerging viral threats, including the global response to the SARS-CoV-2 pandemic in 2020.

### 2020-2024

The development of antivirals targeting SARS-CoV-2 had a record-breaking pace during this period (**Table 1**). Scientists all across the globe have been working hard to find ways to lessen the effects of the virus, develop new vaccinations, and finding new uses for old medications. In order to improve innate immunity against SARS-CoV-2 infections, the present research expanded the spectrum of holistic antiviral techniques by highlighting the relevance of peptide-based antivirals, immunosuppressive drugs, anti-inflammatory molecules, corticosteroids, and natural substances. Additionally, convalescent plasma therapy (CPT)- obtained from previously infected donors who developed antibodies- was further exploited to enhance immune responses in severe COVID-19 cases (Chen et al., 2020). The major challenge in the COVID-19 pandemic was the onset of evolving serotypes and clinical variants across the globe. Therefore, future innovative and diversified antiviral strategies are critically needed for sustained research to combat drug resistance.

Key antiviral agents included Remdesivir (GS-5734), one of the first drugs approved for emergency use, along with other repurposed treatments like Chloroquine, Hydroxychloroquine, Lopinavir/Ritonavir, and Favipiravir, which were tested based on their previous antiviral properties (Dehelean et al., 2020). New molecular candidates were also investigated, such as human monoclonal antibodies (mAbs) 47D11 H2L2-neutralizing Ab, which showed promise in neutralising the SARS-CoV-2 entry in Vero cells with an EC50 of 0.57 μg/mL (Wang et al., 2020). This research opens doors for mAbs that can be used alone or in association with other compounds to treat COVID-19. Peptidomimetic α,β-unsaturated esters, Ebselen, Betulinic acid, and Glycyrrhizic acid were explored for their potential to inhibit viral replication. Various inhibitors targeting the main protease (Mpro) and other essential viral enzymes were developed, including N3, 11a/11b, and Carmofur (Jin et al., 2020; Tiwari et al., 2020). The results of randomised, controlled, and multicenter clinical studies show that immune modulators such as dexamethasone, a corticosteroid, have emerged as essential therapies for decreasing inflammation in severe instances of COVID-19. These treatments have also been shown to considerably reduce death rates in hospitalised patients, according to Recovery 2020 (The RECOVERY Collaborative Group, 2021). Other immunomodulatory agents like tocilizumab, an IL-6 inhibitor, were employed to combat cytokine storms caused by SARS-CoV-2 (Stone et al., 2020; Zeng et al., 2020). The pan-CoV peptide-based fusion inhibitors EK1 and its lipopeptide EK1C4 were also effective against membrane fusion mediated by S proteins of the Omicron subvariants of SARS-CoV-2 and virus-like particle infection. HCoV-OC43’s HR2 region was used to produced peptide EK1 and lipopeptide EK1C4. EK1C4, a lipopeptide with cholesterol and GSGSG-PEG4 linker at its C-terminus, has much higher antiviral effectiveness than EK1.

Several other drugs impairing different stages of viral replication and interfering with nonstructural proteins (NSPs) were explored, such as Camostat Mesylate, Ivermectin, Arbidol Hydrochloride (Umifenovir), Oseltamivir, and Nitazoxanide (Dehelean et al., 2020). Ivermectin of 5 μM concentration inhibited up to 99% of SARS-CoV-2 replication in Vero cells and showed no cytotoxic effects (Caly et al., 2020). The clinical trial studies of Arbidol Hydrochloride (Umifenovir) on COVID-19 patients did not decrease viral load but were still used as an antiviral drug due to its antioxidant potential (Proskurnina et al., 2020). Because vitamin D was also investigated for its ability to strengthen immunological defences, it continued to be used as a medication to treat viral infections (Shalayel et al., 2020).

The year 2021 saw the development and scientific investigation of a number of different antiviral medications in order to combat the worldwide epidemic that was triggered by SARS-CoV-2. Among these medicines, EIDD-2801 (also known as Molnupiravir or MK-4482) has emerged as a potential nucleoside analogue showing considerable activity against SARS-CoV-2 in preclinical models (Wahl et al., 2022). Molnupiravir (EIDD-2801 or MK-4482), which started to treat RNA viruses, has attracted attention for its ability to significantly inhibit SARS-CoV-2 replication (within 48 hours of infection) and transmission, making it a contender for early COVID-19 therapy. Barrigenol, Kaempferol, and Myricetin, extracted from Camelia Sinensis (tea) were discovered in several research (Sharma et al., 2022), also indicated antiviral action. This was mostly due to their capacity to impede viral entrance or reproduction. In order to investigate the therapeutic potential of these plant-based compounds against SARS-CoV-2, the pool of naturally derived medicines that were investigated.

Additionally, throughout the year, continued research was conducted on existing drugs, such as Camostat Mesylate and Ivermectin, to investigate the possibility that these medications may be used to treat SARS-CoV-2 infections. The ability of Camostat Mesylate, a protease inhibitor, to inhibit TMPRSS2, a protein that is necessary for viral entry into host cells, was investigated in this study (Rajter et al., 2021). Likewise, Ivermectin, a standard antiparasitic drug, was examined for its *in vitro* antiviral properties, although clinical outcomes remained ambiguous (Rajter et al., 2021).

As of the year 2021, Monoclonal antibodies (mAbs) continued to be utilised and researched, marking a significant advancement. It was shown that the combination of REGN10933 and REGN10987, which is known as REGN-COV2, was able to successfully lower the viral load and avoid catastrophic outcomes in COVID-19 patients (Weinreich et al., 2021). Monoclonal antibody therapies continued to play a critical role in the treatment landscape, such as Sotrovimab (VIR-7831 or GSK-4182136)/Bebtelovimab (LY-CoV1404‖ LY3853113) and combinations like Tixagevimab/Cilgavimab and Bamlanivimab/Etesevimab remaining at the forefront. The combination of these MAbs effectively neutralised both the wide-type SARS-CoV-2 and the variants that are now causing concern, including Alpha (B.1.1.7), Beta (B.1.351), Gamma (P.1), and Delta (B.1.617.2). Additionally, they continued to have significant action against Omicron lineages, such as BA.2.75, BA.4, BA.4.6, and BA.5.

Other monoclonal antibody therapies, such as Casirivimab and Imdevimab, Sotrovimab, and Bamlanivimab with Etesevimab, were also evaluated and used as therapeutic interventions in high-risk patients. The investigation into repurposed drugs persisted in 2021, with compounds like Darunavir (an HIV protease inhibitor), Nitazoxanide (an antiparasitic), and Sorafenib (a cancer therapy) being subjected to clinical trials to assess their antiviral potential (Sun et al., 2021). In addition, Foretinib, Eprosartan, and Inarigivir soproxil were investigated for their capacity to suppress the replication of SARS-CoV-2 and to reduce the severity of the symptoms of infection (Joshi and Poduri, 2021). Dexamethasone and other more recent medications, such as Sarilumab, an IL-6 receptor antagonist, continued to be administered to strengthen the role that immunological modulation performs well against severe COVID-19 (Castelnovo et al., 2021). The findings highlighted the importance of addressing hyperinflammatory syndrome and acute respiratory failure, conditions that were often associated with unfavourable outcomes in patients with severe COVID-19. Innovative agents like P2C-1F11, a neutralising antibody, and CR3022, a monoclonal antibody aimed at the receptor-binding domain of SARS-CoV-2, have offered fresh perspectives on the efficacy of antibody-based therapies in the prevention or treatment of SARS-CoV infections (Atyeo et al., 2021). Finally, naturally derived chemicals such as Aranotin, Ajmalicine, Silymarin, and Ursonic Acid were investigated for their antiviral effects, increasing interest in plant-based and naturally occurring substances for medicinal development against SARS-CoV-2 (Kumar et al., 2021).

The breakthrough development of antiviral drugs for SARS-CoV-2 in 2022 was oral antiviral medicine Paxlovid, a combination of Nirmatrelvir (PF-07321332) with Ritonavir, which emerged as a prominent antiviral therapy for the treatment of COVID-19. This oral treatment significantly reduced the risk of hospitalisation and severe outcomes in high-risk patients when administered early in infection. The Paxlovid leverages the main protease (Mpro) inhibition by Nirmatrelvir and utilises Ritonavir to enhance its pharmacokinetics, with mild side effects. Nirmatrelvir was equally effective as remdesivir, which has been shown to reduce hospitalisation or mortality by 87% in patients with severe COVID-19 (Gottlieb et al., 2022). The efficacy of Paxlovid in lowering viral load established it as a cornerstone in the treatment of COVID-19 arsenal against COVID-19. This highlights a combination therapy trend in antiviral development toward optimising existing medications. Continuing the trend of repurposing existing drugs, the combination of parenteral polymerase inhibitor Remdesivir and Molnupiravir for oral treatment of COVID-19 in non-hospitalised patients was extensively evaluated in 2022. Remdesivir, an already established antiviral agent, demonstrated its ability to reduce the duration of hospitalisation in patients with severe COVID-19.

Inotodiol, neosarcodonin, Dutasteride, Etoposide, and Golvatinib were explored for their potential antiviral properties. Inotodiol, a triterpenoid derived from *Inonotus obliquus* (I. obliquus), a wild Chaga mushroom, was one of the new compounds whose antiviral and anti-inflammatory efficacy against SARS-CoV-2 was also investigated. Similarly, the well-established anti-inflammatory qualities of neosarcodonin A, B, and C—isolated from *Sarcodon scabrosus* bitter mushroom—led to its repurposing in light of the rising interest in alternate sources of antiviral compounds. The function of antiandrogens as agents of protection against COVID-19 was also investigated. Under this particular scenario, a double-blind, placebo-controlled randomised clinical trial (RCT) was conducted for dutasteride (DUTA) with early antiandrogen therapy (EAT) was recommended as a treatment for COVID-19 (EAT-DUTA AndroCoV). Etoposide, a medication that contains epipodophyllotoxin, has been used to treat immune-mediated inflammatory illnesses linked to cytokine storm syndrome for over 40 years. This molecule has been investigated for COVID-19 treatment. The most significant binding free energy is exhibited by golvatinib (IC50 around 1uM), indicating that it may be evaluated for anti-SARS-CoV-2 infection *in vitro* (Shen et al., 2023).

Simeprevir, a protease inhibitor and Paritaprevir authorised for managing HCV infection, has shown potential antiviral efficacy at EC50 of 1.41 ± 0.12 μM to suppress SARS-CoV-2 replication in Vero E6 cells (Muturi et al., 2022). Ivermectin, tipranavir, and paritaprevir have dose-dependent inhibitory action (IC50) against protease (3CLPro) at 21.5, 27.7, and 73.4 µM, indicating their potential as anti-SARS-CoV-2 inhibitors (Mody et al., 2021). Furthermore, *in silico* investigations on alpha-ergocryptine and diosgenin glucoside revealed inhibitory ability against SARS-CoV-2 endoribonuclease (NSP15) and potentially bioactive molecules.

Throughout year 2023, researchers found and analysed a wide range of compounds, demonstrating the changing landscape of antiviral development. Prominent among the antiviral agents in 2023 are combinations of drugs such as bamlanivimab/etesevimab, casirivimab/imdevimab, and tixagevimab/cilgavimab have been actively researched and administered to enhance the efficacy of COVID-19 treatment, especially in high-risk patients. Remdesivir, Paxlovid (nirmatrelvir/ritonavir), and molnupiravir are well-known medicines still important in COVID-19 care. In addition to these proven drugs, the year saw the development of many new compounds having antiviral characteristics. Natural compounds such as cafestol, kahweol, and theaflavin 3,3′-digallate showed inhibitory potential in suppressing SARS-CoV-2 replication. Other potential possibilities were tested for antiviral efficacy, including dihydroergocristine, ergometrine, and ubidecarenone. The year also witnessed a concerted effort to better understand the immunomodulatory landscape of COVID-19. This effort included the development of several interleukin-6 inhibitors (such as tocilizumab and salirumab) and other antiviral drugs (such as Nakinra, baricitinib, and tofacitinib) in order to develop treatments that would reduce the severity of the disease.

As of 2024, the prevalence of clinical variations of concern (VoC) of SARS-CoV-2 in the U.S. renders them resistant to several neutralizing monoclonal antibodies (mAbs). The US FDA is now not allowing or is revoking several products, including REGEN-COV (casirivimab and imdevimab), Sotrovimab, Bamlanivimab and Etesevimab, Bebtelovimab, and Evusheld (tixagevimab/cilgavimab) as of the end of 2023. They cannot be used for pre-exposure prophylaxis to prevent or treat COVID-19 under the EUA. Meanwhile, several existing molecules have received FDA approval for COVID-19 treatment, including tocilizumab administered intravenously or subcutaneously, remdesivir as an intravenous therapy, baricitinib as an oral immune modulator, and Paxlovid (nirmatrelvir/ritonavir) as an oral antiviral treatment for adults and long-COVID patients. Tocilizumab is an intravenous or subcutaneous immune modulator for hospitalized children aged 2 to under 18 years. Vilobelimab is an intravenous infusion immune modulator approved for treating hospitalized adults, provided it is administered within 48 hours of initiating invasive mechanical ventilation (IMV) or extracorporeal membrane oxygenation (ECMO). Anakinra is an immune modulator approved for the treatment of COVID-19 in hospitalized adults with pneumonia who require supplemental oxygen, whether low- or high-flow and are at risk of developing severe respiratory failure. Molnupiravir is an oral antiviral that has received FDA authorization for emergency use in treating mild-to-moderate COVID-19 in individuals aged 18 and older at high risk for progression to severe COVID-19. Baricitinib is an oral immune modulator approved for emergency use in hospitalized children aged 2 to under 18 years who require supplemental oxygen, invasive mechanical ventilation, or extracorporeal membrane oxygenation (ECMO) due to COVID-19. Ensitrelvir, a 3C-like protease inhibitor, when administered orally once a day for five days, dramatically decreased viral RNA levels by day 4 and showed a significantly quicker resolution of the five typical symptoms of the Omicron SARS-CoV2 (Yamato et al., 2024). Based on such evidences, Ensitrelvir received emergency approval in Japan in 2022. However, larger trials are required for further confirmation.

## Conclusion

As of now, the nucleoside analogues Ribavirin, Favipiravir, and Remdesivir; the HIV-1 protease inhibitors lopinavir and Ritonavir; and the SARS-CoV-2 main protease (Mpro/3CLpro) inhibitor Nirmatrelvir stand out as the pivotal antiviral agents based on their track record of use and effectiveness in coronavirus outbreaks. Despite how well these antivirals generally work in clinical settings, much work still needs to be done to make an antiviral that works well against coronaviruses.
